# Supplementary material for: Identification of photoperiod-induced specific miRNAs in the adrenal glands of Sunite sheep (Ovis aries)
Source: Front Vet Sci. 2022 Jul 22;9:888207. doi: 10.3389/fvets.2022.888207 (PMC9354845; doi:10.3389/fvets.2022.888207)
Supplement: Supplementary file 1 [file Data_Sheet_1.ZIP › Supplementary file/miRDeep2 file.zip!/miRDeep2 file/Novel_Structure/SPLP42A3/Novel_300.pdf]

Provisional ID	:	NC_019477.2_32472
Score total	:	1
Score for star read(s)	:	-1.3
Score for read counts	:	0
Score for mfe	:	0.7
Score for randfold	:	1.6
Score for cons. seed	:	
Total read count	:	38
Mature read count	:	38
Loop read count	:	0
Star read count	:	0

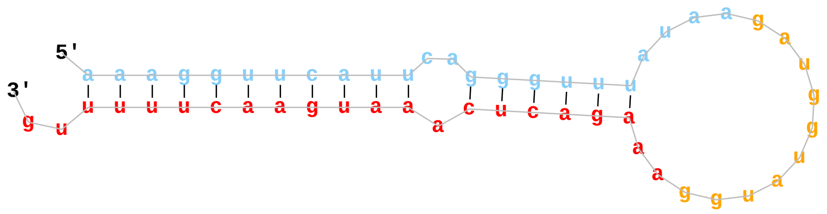

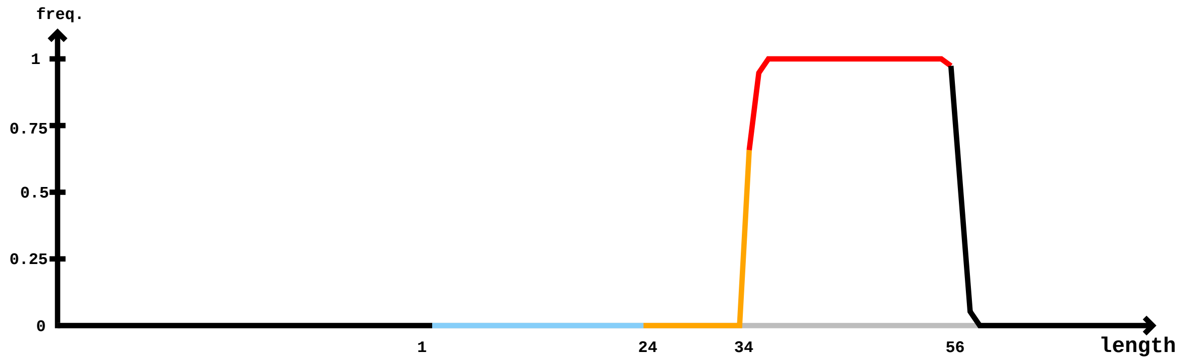


```
                                            Star                                     Mature
5'- aggugguuaauagccauuauauaaauuaugcuggacaaaagguucauucaggguuuauaagaugguauggaaagacucaaaugaacuuuuuggucagcgcaauagaaagcaa   -3'    exp
    .(((((((...))))))))...........((((..(((((((((((((..((((((................)))))).)))))))))))))..))))((........))..    reads      mm          sample
    ......................................................................aaagacucaaaugaacuuuuug....................    1          0           seq
    ......................................................................aaaAacucaaaugaacuuuuug....................    24         1           seq
    .......................................................................aagacCcaaaugaacuuuuu.....................    1          1           seq
    .......................................................................aaAacucaaaugaacuuuuug....................    10         1           seq
    ........................................................................aAacucaaaugaacuuuuuggu..................    2          1           seq
```
